# Cytotoxicity Effect of Iron Oxide (Fe_3_O_4_)/Graphene Oxide (GO) Nanosheets in Cultured HBE Cells

**DOI:** 10.3389/fchem.2022.888033

**Published:** 2022-05-09

**Authors:** Yule Zhang, Yatian Zhang, Zhijin Yang, Yan Fan, Mengya Chen, Mantong Zhao, Bo Dai, Lulu Zheng, Dawei Zhang

**Affiliations:** ^1^ Engineering Research Center of Optical Instrument and System, the Ministry of Education, Shanghai Key Laboratory of Modern Optical System, University of Shanghai for Science and Technology, Shanghai, China; ^2^ Medical College Jining Medical University, Jining, China; ^3^ Department of Physics and Electronic Engineering, Heze University, Heze, China; ^4^ Shanghai Institute of Intelligent Science and Technology, Tongji University, Shanghai, China

**Keywords:** Fe_3_O_4_/GO nanosheets, cytotoxicity effects, oxidative stress, Ca^2+^ influx, apoptosis

## Abstract

Iron oxide (Fe_3_O_4_), a classical magnetic material, has been widely utilized in the field of biological magnetic resonance imaging Graphene oxide (GO) has also been extensively applied as a drug carrier due to its high specific surface area and other properties. Recently, numerous studies have synthesized Fe_3_O_4_/GO nanomaterials for biological diagnosis and treatments, including photothermal therapy and magnetic thermal therapy. However, the biosafety of the synthesized Fe_3_O_4_/GO nanomaterials still needs to be further identified. Therefore, this research intended to ascertain the cytotoxicity of Fe_3_O_4_/GO after treatment with different conditions in HBE cells. The results indicated the time-dependent and concentration-dependent cytotoxicity of Fe_3_O_4_/GO. Meanwhile, exposure to Fe_3_O_4_/GO nanomaterials increased reactive oxygen species (ROS) levels, calcium ions levels, and oxidative stress in mitochondria produced by these nanomaterials activated Caspase-9 and Caspase-3, ultimately leading to cell apoptosis.

## Introduction

Fe_3_O_4_ nanoparticles (Fe_3_O_4_ NPs) are also a classical magnetic substance, which have attracted increasing attention because they have been successfully approved by the Food and Drug Administration for use in MRI (Chen et al., 2011; Wu et al., 2021). Currently, Fe_3_O_4_ NPs are frequently used in MRI, biological separation, hyperthermia therapy, and other biomedical fields.

As another interesting nanocomponent commonly employed in drug delivery, GO has hydrophilic and hydrophobic oxygen-containing functional groups, like hydroxyl, carboxyl, and epoxy groups, making it easily soluble in water and various organic solvents (Metin et al., 2014; Tang et al., 2018). Its unusual properties, including electrical, optical, thermal, and mechanical properties, are predominantly determined by the chemical structure of the Sp3 carbon domain surrounded by the Sp2 carbon domain (Zakharova et al., 2021). On the other hand, GO has another critical feature of its structure with a large specific surface area. GO has become the focus of widespread interest in the field of materials over the past few years because of its unique thermal, electronic, and optical properties, and high drug delivery rate of up to 200% (Vannozz et al., 2021). Therefore, GO-based nanocomposites have aroused extensive attention in the biomedical field, especially in the diagnosis and treatment of tumors.

Mounting literature indicated that the GO coupled with magnetic nanoparticles could serve as a potential material for the diagnosis and treatment of cancers (Chen H. et al., 2021). Currently, several researches reported various methods to synthesize magnetic and graphite nanostructured composites (Fe_3_O_4_/GO) for catalytic, water purification, biomedical diagnostic, and therapeutic applications (Jedrzejczak-Silicka, 2017; Yan et al., 2019; Niu et al., 2021; Sadighian et al., 2021). This combination of topical hyperthermia materials is also regarded as a promising candidate for drug delivery (Karimi and Namazi, 2021; Wen et al., 2021). Nowadays, some of these attractive metal oxides have been documented to be cytotoxic and genotoxic, potentially leading to the destruction of mitochondrial membrane integrity, DNA fragmentation, and cell death (Li et al., 2018). Due to the tremendous potential of Fe_3_O_4_/GO in biomedical and other fields, recent researches have focused on the potential cytotoxicity and genetic toxicity of these hybrids (Ahamed et al., 2020; Zhang H. et al., 2020). However, the relationship of Fe_3_O_4_/GO exposure with ROS and calcium ion levels and apoptosis remains enigmatic. Advances in understanding the relationship between physicochemical parameters and the potential cytotoxic impacts of synthetic hybrids, including these analyses, need to be clarified and should correspond to mainstream nanotechnology and its wide range of biomedical applications (Qiang et al., 2021). Therefore, this study set out to evaluate cellular responses, including ROS levels, calcium ion levels, mitochondrial superoxide levels, and apoptosis levels of human bronchial epithelial (HBE) cell lines, after Fe_3_O_4_/GO exposure.

As reported, calcium influx can activate Caspase-9 to facilitate the cleavage of Caspase-3 and activate Caspase-3, contributing to cell apoptosis (Valdiglesias, 2022; Ayse Kaplan et al., 2019; Chen et al., 2005). In this study, we investigated the cytotoxicity of Fe_3_O_4_/GO after incubation with HBE cells. The results manifested that exposure to Fe_3_O_4_/GO with high concentration increased ROS levels, Ca^2+^ influx, and mitochondrial dysfunction and then induced apoptosis. It was verified that nanomaterial exposure augmented oxidative stress, calcium influx, and mitochondrial superoxide generation, which promoted the activation of Caspase-9/Caspase-3, ultimately resulting in cell apoptosis.

## Experimental Section

### Materials and Reagents

All chemical reagents for synthetic materials were obtained from Sinopharm Chemical Reagent Co. Reagents used in cell culture such as phosphate-buffered saline (PBS), Dulbecco’s modified Eagle’s medium (DMEM), fetal bovine serum (FBS), penicillin and streptomycin were provided by Gibco, Invitrogen. Cell count kit-8 (cck-8) and Fluo-4AM were purchased from Beyotime Biotechnology. 2'7'-dichlorofluorescein diacetate (DCFH-DA), Mitosox red, and 4', 6-diamidine-2-phenylindole dihydrochloride (DAPI) were provided by Sigma–Aldrich. Antibodies of Caspase-9/Caspase-3 were obtained from a protein technology company. The secondary antibody used Alexa Fluor 488 conjugated goat anti-mouse and Cy3 conjugated goat anti-rabbit, which were purchased from Servicebio. Calcein-AM/PI and Annexin-V/PI double staining kits were purchased from Dojindo laboratories.

### Synthesis and Characterization of Materials

#### Synthesis of Fe_3_O_4_/GO

In order to produce Fe_3_O_4_/GO, glycine is used as a linker. First, 20 mg Fe_3_O_4_ nanospheres were dispersed in 0.5 mg/ml water, ultrasonic treatment until uniform dispersion, and then functionalized with glycine so that the -NH_2_ group was attached to its surface. 20 mg GO sample was ultrasonically stripped in 60 ml H_2_O to generate a homogeneous GO aqueous suspension. The carboxyl groups on the surface of GO were then activated by 8 mg N-hydroxysuccinimide (NHS) and 10 mg 1-(3-dimethylaminopropyl-1)-3-ethylcarbondiimide (EDC). The mixture of modified Fe_3_O_4_ and GO was stirred for 2 h, and the resulting product was centrifuged, washed with water and ethanol several times, and dried at 100°C.

#### Characterization of Fe_3_O_4_/GO

The images of NP morphology were obtained using a transmission electron microscope (TEM; Philips/FEI Company CM300 FEG-ST). Fourier infrared spectroscopy (FTIR) was analyzed by IRTracer-100, Japan. Hydrodynamic sizes of Fe_3_O_4_/GO were evaluated by dynamic light scattering (DLS) via Malvern (Zetasizer Nano S90) in water and DMEM, respectively. The Zeta potential of Fe_3_O_4_/GO was analyzed by Malvern, Zetasizer Nano S90.

#### Cell Culture

HBE cells and Ad12-SV40 2B (BEAS-2B) cells were obtained from American Type Culture Collection (ATCC, United States). Cells were cultured in DMEM containing 10% FBS and 1% penicillin and streptomycin at 37°C with 5% CO_2_.

### Cytotoxicity Detection After Fe_3_O_4_/GO Stimulations

Cell viability was measured by cck-8. HBE cells and BEAS-2B cells were seeded in 96-well plates overnight and Fe_3_O_4_/GO in DMEM was added to each well at a dose of 0, 10, 20, 50, 100, and 200 μg/ml for 3 replicates per group. HBE cells were tested after 6, 12, 24, and 48 h of co-incubation and BEAS-2B cells were tested after 12, 24 h of co-incubation later according to the manufacturer’s instructions.

### Oxidative Stress Detection After Fe_3_O_4_/GO Stimulations

Oxidative stress changes are represented by reactive oxygen species (ROS). The fluorescence intensity of DCFH-DA is the most commonly used method to detect intracellular ROS levels. HBE cells were seeded in confocal dishes overnight. Fe_3_O_4_/GO (0, 100, 200 μg/ml) in DMEM was added and then coincubated after 24 h. Then, it was cleaned with PBS three times, then prepared DCFH-DA staining solution was added and incubated for 15 min. It was cleaned with PBS three times, observed, and analyzed by using a confocal microscope (LSM 900, ZEISS, Germany) (Ex: 505 nm Em: 525 nm).

Mitosox red is a mitochondrial superoxide indicator. HBE cells were seeded in confocal dishes overnight. Fe_3_O_4_/GO (0, 100, 200 μg/ml) in DMEM was added and then coincubated after 24 h. Then, it was cleaned with PBS three times, prepared Mitosox red and DAPI staining solution was added and incubated for 15 min. It was cleaned with PBS three times, observed, and analyzed *via* a confocal microscope (LSM 900, ZEISS, Germany) (Mitosox red, Ex: 510 nm Em: 580 nm; DAPI, Ex: 350 nm Em: 461 nm).

### Ca^2+^ Levels Detection After Fe_3_O_4_/GO Stimulations

Ca^2+^ levels were detected by Fluo-4AM. HBE cells were seeded in confocal dishes overnight. Fe_3_O_4_/GO (0, 100, 200 μg/ml) was added and then co-incubated for 24 h. Then, it was cleaned with PBS three times and then prepared Fluo-4AM staining solution was added, and incubated for 15 min. It was cleaned with PBS three times, observed, and analyzed under a confocal microscope (LSM 900, ZEISS, Germany) (Ex: 494 nm Em: 516 nm).

### Dead/Live Cells Detection After Fe_3_O_4_/GO Stimulations

Dead/live cells were analyzed by using a calcein-AM/PI double staining kit. HBE cells were seeded in confocal dishes overnight. Fe_3_O_4_/GO (0, 100, 200 μg/ml) in DMEM was added and then coincubated for 24 h. Then, it was cleaned with PBS three times and prepared calcein-AM/PI staining solution was added and incubated for 15 min. It was cleaned with PBS three times, observed, and analyzed under a confocal microscope (LSM 900, ZEISS, Germany) (Calcein-AM, Ex: 490 nm Em: 515 nm; PI, Ex: 530 nm Em: 617 nm).

### Apoptosis Test

HBE cells were seeded in confocal dishes overnight. Fe_3_O_4_/GO (0, 100, 200 μg/ml) in DMEM was added and then coincubated for 24 h. Then, cells were stained with Annexin-V FITC/PI and analyzed by confocal microscope. (LSM 900, ZEISS, Germany) (Annexin-V FITC, Ex: 488 nm Em: 515 nm; PI, Ex: 530 nm Em: 617 nm).

### Immunofluorescence Staining

HBE cells were seeded in confocal dishes overnight. Fe_3_O_4_/GO (200 μg/ml) in DMEM was added and then co-incubated for 24 h. Then, cells were immunofluorescence stained by Caspase-3 antibody and Caspase-9 antibody and observed under a confocal microscope (LSM 900, ZEISS, Germany).

## Results and Discussion

### Characterization of Fe_3_O_4_/GO

**FIGURE 1:**
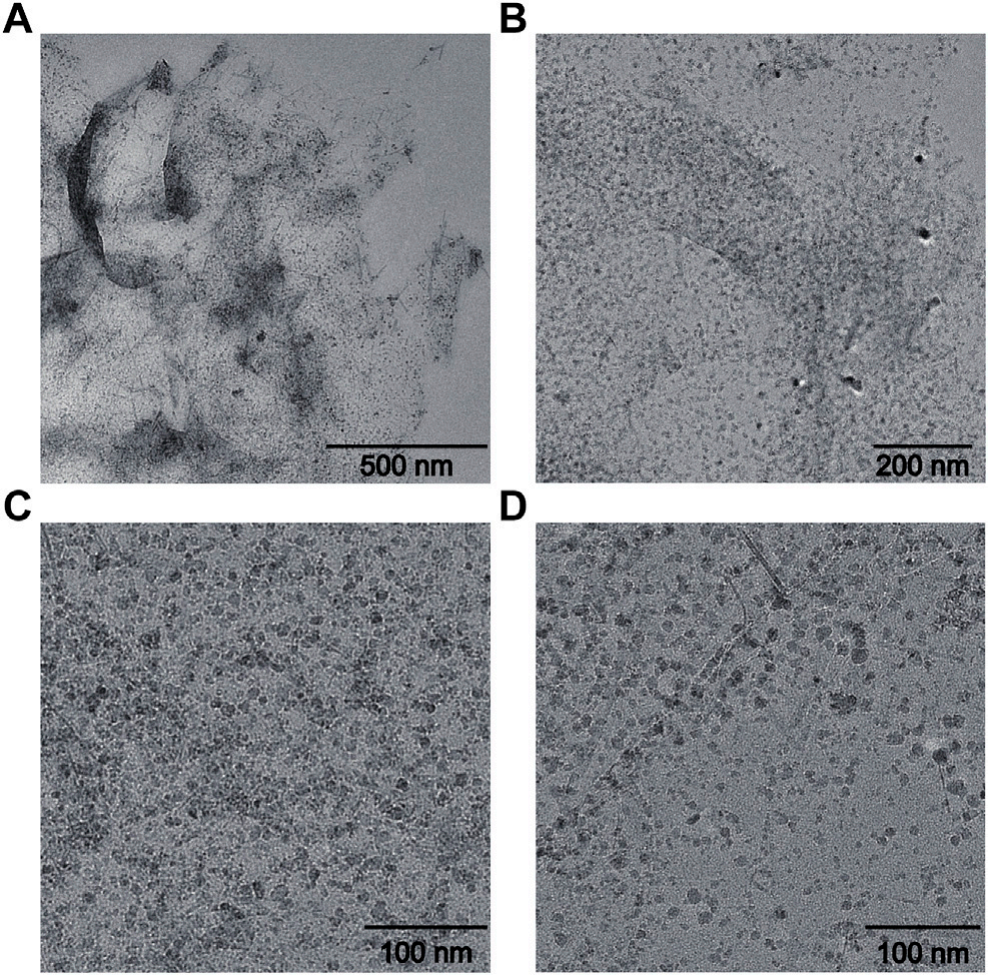
TEM characterization of Fe_3_O_4_/GO. **(A)** scale bar: 500 nm, **(B)** scale bar: 200 nm, **(C–D)** scale bar: 100 nm.

It could be found that GO showed transparent sheet-like gauze with folds at the edge of the sheet. The research of Ajayan et al. suggested that folding was majorly attributable to the destruction of the C=C double bond caused by Sp2 hybrid oxygen-containing functional groups on the graphite oxide (Datta et al., 2018; Sadighian et al., 2021). Fe_3_O_4_ particles prepared by a coprecipitation method have a diameter of approximately 10 nm. However, due to the interaction between coulomb force and van der Waals force among the nanoparticles, a few of the nanoparticles exhibit the agglomeration phenomenon (Dyer et al., 2015; Fu and Li, 2014). Therefore, some Fe_3_O_4_ nanoparticles in the composite have a particle size of 30–50 nm after agglomeration in Figure 1 (Narayanaswamy and Srivastava, 2017). However, the overall dispersion is favorable.

**FIGURE 2:**
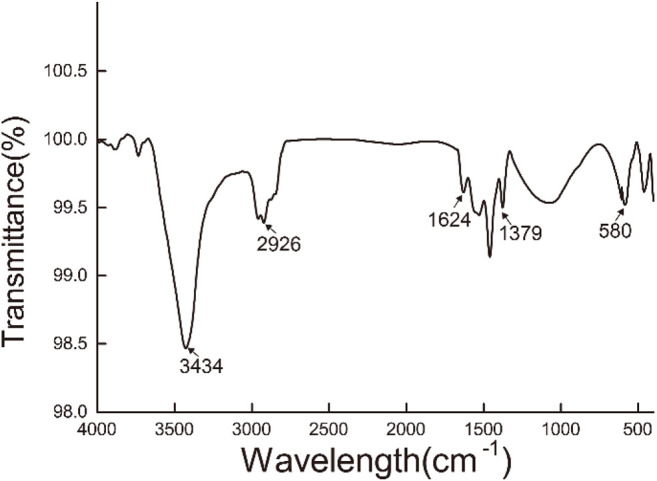
FTIR of Fe_3_O_4_/GO.

As displayed in Figure 2, Fourier transform infrared spectroscopy of Fe_3_O_4_/GO was analyzed. The absorption peak at 3,434 cm^−1^ was attributed to the stretching vibration of OH from GO, and the absorption peak near 2,926 cm^−1^ was attributed to the stretching vibration of CH_2_ from synthetic Fe_3_O_4_/GO (Hanh et al., 2018). The absorption peak at 1,624 cm^−1^ was accounted for by the C=O stretching vibration at the GO edge (Narayanaswamy and Srivastava, 2017). The absorption peak at 1,379 cm^−1^ was caused by the C-O-C stretching vibration on the GO surface (Seyyed et al., 2021). The absorption peak at 580 cm^−1^ was induced by the stretching vibration of Fe-O-Fe (Zhang et al., 2017). In summary, it was indicated that Fe_3_O_4_/GO nanoparticle complex with high purity was prepared.

**FIGURE 3:**
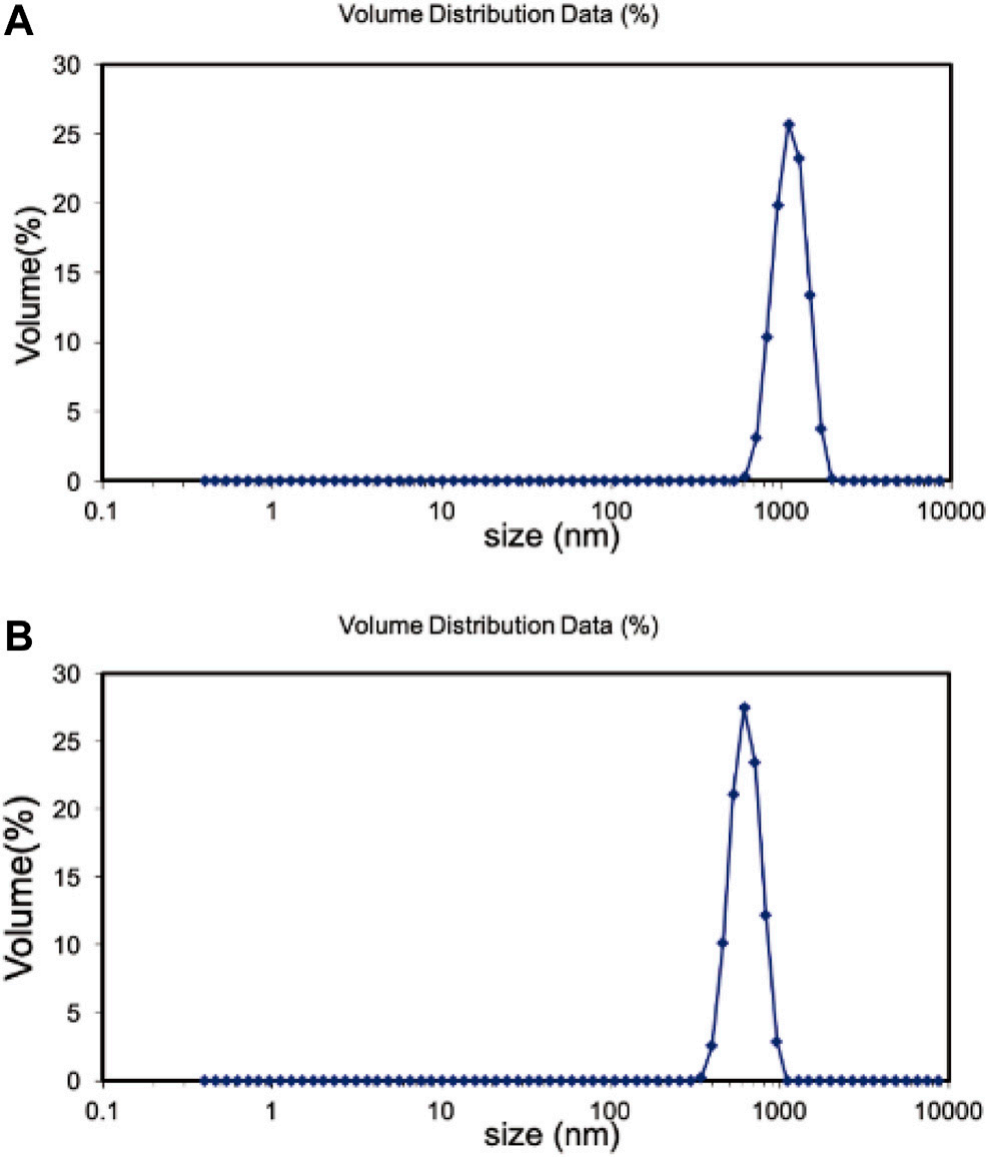
**(A)** Hadrodynamic sizes (nm) of Fe_3_O_4_/GO in dH_2_O. **(B)** Hadrodynamic sizes (nm) of Fe_3_O_4_/GO in DMEM.

**FIGURE 4:**
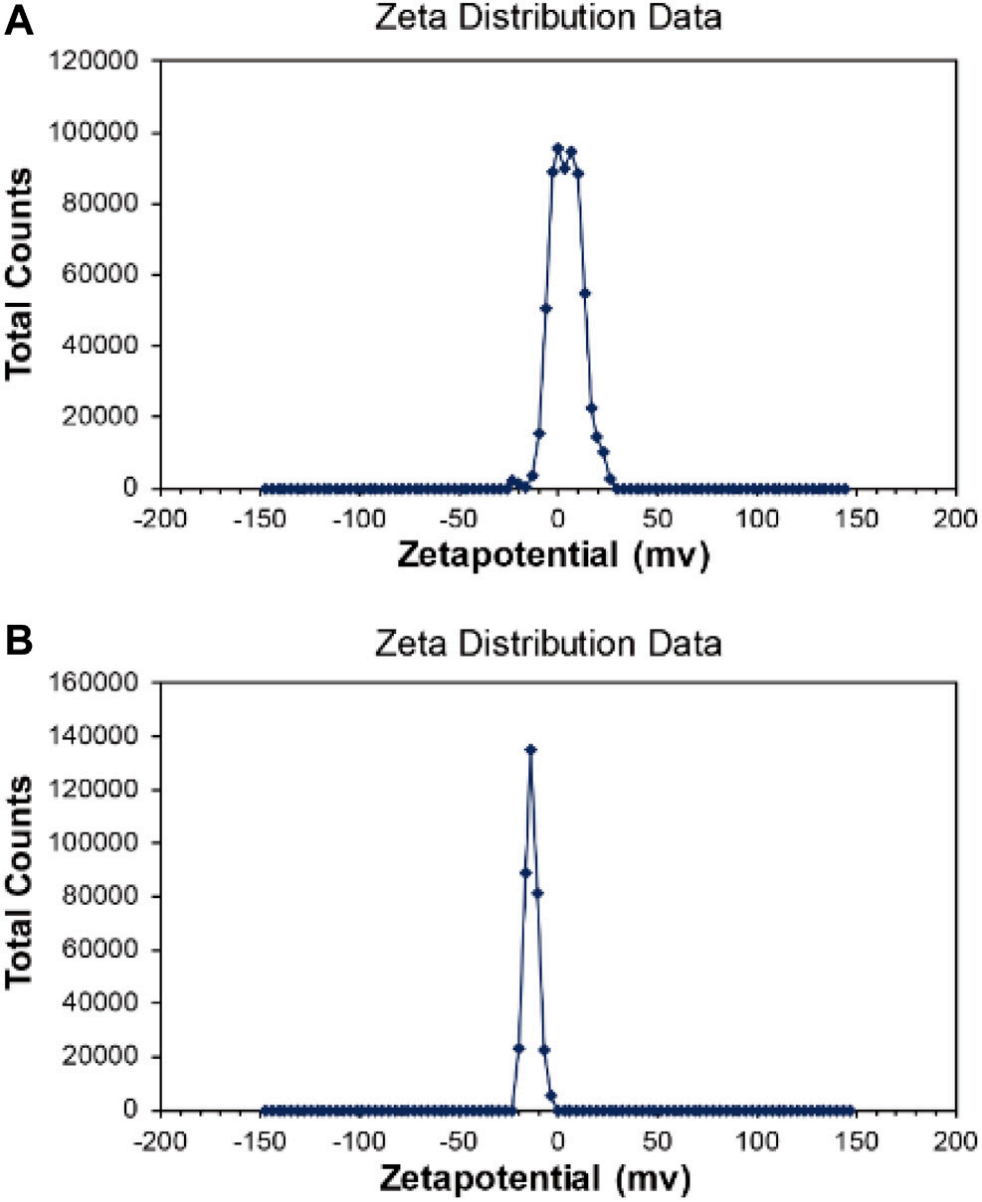
**(A)** Zeta potential of Fe_3_O_4_/GO in dH_2_O. **(B)** Zeta potential of Fe_3_O_4_/GO in DMEM.

The hydration particle size of Fe_3_O_4_/GO was analyzed in deionized (DI) water and DMEM, respectively by DLS. As shown in Figure 3A, Fe_3_O_4_/GO was 1,325 nm in dH_2_O. There is a certain aggregation in DI water, so the measured particle size is larger. Due to the presence of serum in the medium, the serum protein such as albumin could form a corona which stabilization of the materials in the suspension (Wang et al., 2016). Thus, Fe_3_O_4_/GO had better dispersion in the medium with a particle size of 1,164 nm in Figure 3B. The Zeta potential of materials was in the range of 3.96 mV and −13.6 mV in DI water and DMEM, respectively, which changes the positive charge in DI to negative charge in DMEM (Figure 4).

### Cytotoxicity Effect of Fe_3_O_4_/GO

**FIGURE 5:**
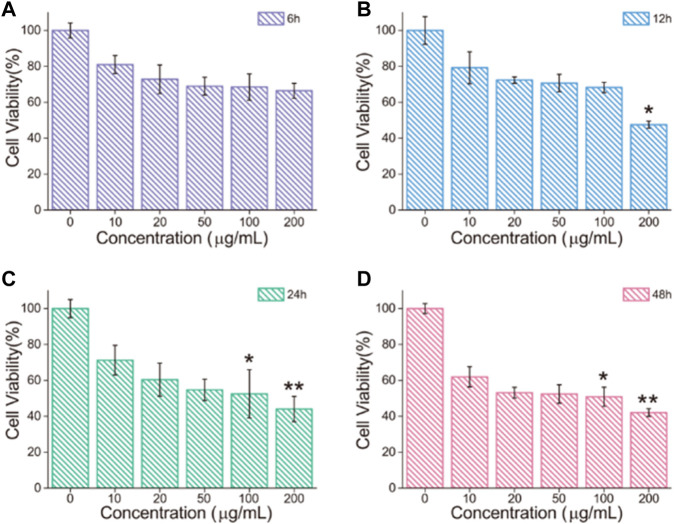
**(A–D)** HBE cell viability after treatment with different concentrations of Fe_3_O_4_/GO for 6, 12, 24 and 48 h, respectively, (*p < 0.05, **p < 0.01, ***p < 0.001).

It was evaluated at different concentration and different times of Fe_3_O_4_/GO cytotoxicity *via* cck-8 assay, respectively. HBE cells were cultured with different concentrations (0, 10, 20, 50, 100, and 200 μg/ml) of Fe_3_O_4_/GO, and cell viability was detected after 6, 12, 24, and 48 h by cck-8 assay. As shown in Figure 5A, the cell survival rate still reached more than 60% after 6 h Fe_3_O_4_/GO exposure at 200 μg/ml. In short periods, even high concentrations of Fe_3_O_4_/GO can have a rational biosafety profile. After 12 h, we can significantly infer that cell viability was decreased to 47.55% after the highest concentration of Fe_3_O_4_/GO exposure shown in Figure 5B. Compared with Figure 5A, the results indicated that cytotoxicity of Fe_3_O_4_/GO was time-dependent. In Figures, 5C,D, HBE cell viability was only around 40% after 24 and 48 h Fe_3_O_4_/GO stimulation at the concentration of 200 μg/ml. It is worth noting that cell viability was less than 60% after 48 h, even at low concentrations (20 μg/ml). However, other reports indicated that these nanoparticles had the potential to produce toxic effects in cells, and their toxic effects were related to their size, concentration, time, shape, and the cell type (Wang et al., 2020; Zhang S. et al., 2020). Thus, we also verified the cytotoxicity effects of Fe_3_O_4_/GO in BEAS-2B cells, which also belong to human lung epithelial cells. As shown in Supplementary Figure S1, with an increasing concentration of Fe_3_O_4_/GO, the BEAS-2B cell’s cytotoxicity was Fe_3_O_4_/GO nanosheet concentration-dependent and time-dependent after 12 and 24 h co-incubation. This study showed that the cytotoxicity effects of Fe_3_O_4_/GO on cells was time-dependent and concentration-dependent.

### Oxidative Stress Analysis of Cells After Fe_3_O_4_/GO Nanoparticle Exposure

**FIGURE 6:**
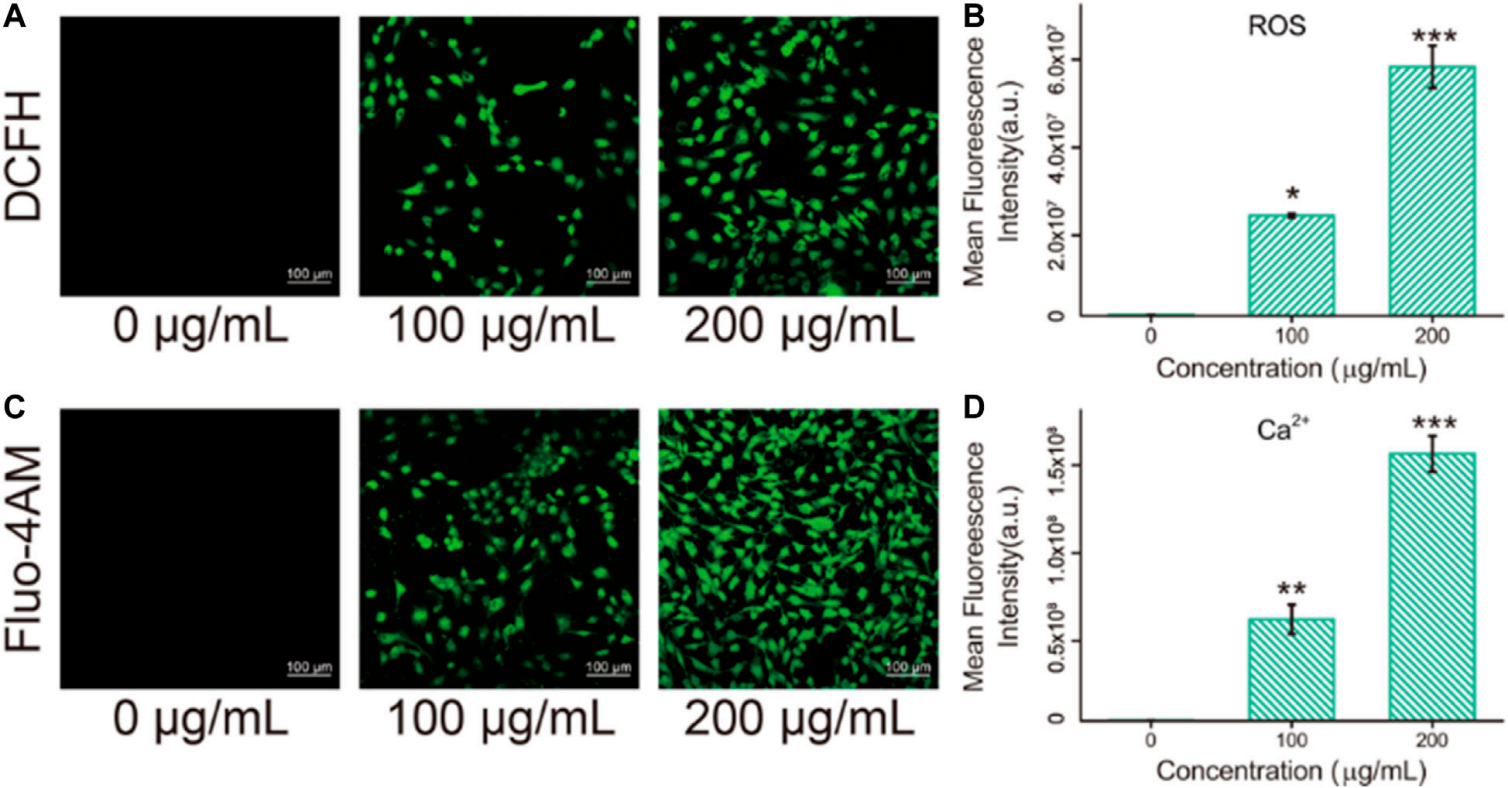
**(A)** ROS levels in HBE cells were detected by DCFH after co-incubation with different concentration of Fe_3_O_4_/GO after 24 h (scale bar: 100 μm). **(B)** Mean fluorescence intensity of DCFH after co-incubation for 24 h. **(C)** Ca^2+^ levels in HBE cells were detected by Fluo-4AM after co-incubation with different concentration of Fe_3_O_4_/GO after 24 h (scale bar: 100 μm). **(D)** Mean fluorescence intensity of Fluo-4AM after co-incubation for 24 h.

The imbalance between oxidants and antioxidants favors oxidants and may culminate in so-called “oxidative stress” (Salvador et al., 2021). As a product of oxidative stress reaction, ROS produced by the interaction between nanomaterials and cells has been reported as one of the pivotal causes of cell damage. It has been previously reported by several researchers that ROS [such as superoxide anions (O_2_•^−^), hydroxyl radicals (HO•), and hydrogen peroxide] levels could enhance in human cells after exposure to Fe_3_O_4_/GO nanosheets. Literature also unravels that iron oxide nanoparticles can induce cytotoxicity by activating oxidative stress responses (Ahamed et al., 2020). To further detect whether Fe_3_O_4_/GO induces ROS production in HBE cells, DCFH staining was used. Oxidative stress levels were expressed as ROS levels and detected by DCFH fluorescent probe. As shown in Figure 6A, it can be found that the green fluorescence (DCFH fluorescent probe) in the cells increased significantly in response to Fe_3_O_4_/GO concentration after 24 h of co-incubation, indicating that the oxidative stress level of the cells increases significantly after Fe_3_O_4_/GO stimulation. In addition, our quantitative data (Figure 6B) showed that ROS levels in the Fe_3_O_4_/GO group were significantly higher than in other control group in a concentration-dependent manner. These results suggested that Fe_3_O_4_/GO induced ROS production and resulted in oxidative stress in HBE cells.

### Calcium Influx of Cells After Fe_3_O_4_/GO Exposure

Fluo-4AM was used to detect the calcium ion levels in cells by means of confocal observation. As shown in Figures 6C, D, fluorescence intensity enhanced significantly with the increase of concentration after coincubation with Fe_3_O_4_/GO for 24 h. Most studies elucidated that ROS in the mitochondrial respiratory chain could elevate Ca^2+^ levels (Zhang et al., 2022). Therefore, intracellular free Ca^2+^ may be implicated in the mechanisms of apoptosis (Zhang et al., 2015). In this study, it was confirmed that Fe_3_O_4_/GO induced ROS generation and Ca^2+^ influx in HBE cells. Considering that calcium signaling is a crucial manipulator of cell function, ER is the dominant source of intracellular calcium and assumes an essential role in the process of cell apoptosis. Following Fe_3_O_4_/GO exposure, intracellular calcium levels were increased by releasing Ca^2+^ of ER. The disruption of intracellular calcium homeostasis contributes to calcium metabolism disorders and impairs protein folding. The long-term accumulation of misfolded proteins in ER results in ER stress-mediated apoptosis. Thus, we can infer that Fe_3_O_4_/GO may lead to HBE apoptosis via ROS-induced Ca^2+^ influx, which was verified in subsequent experiments.

### Mitochondrial Superoxide Levels of Cells Analysis After Fe_3_O_4_/GO Exposure

**FIGURE 7:**
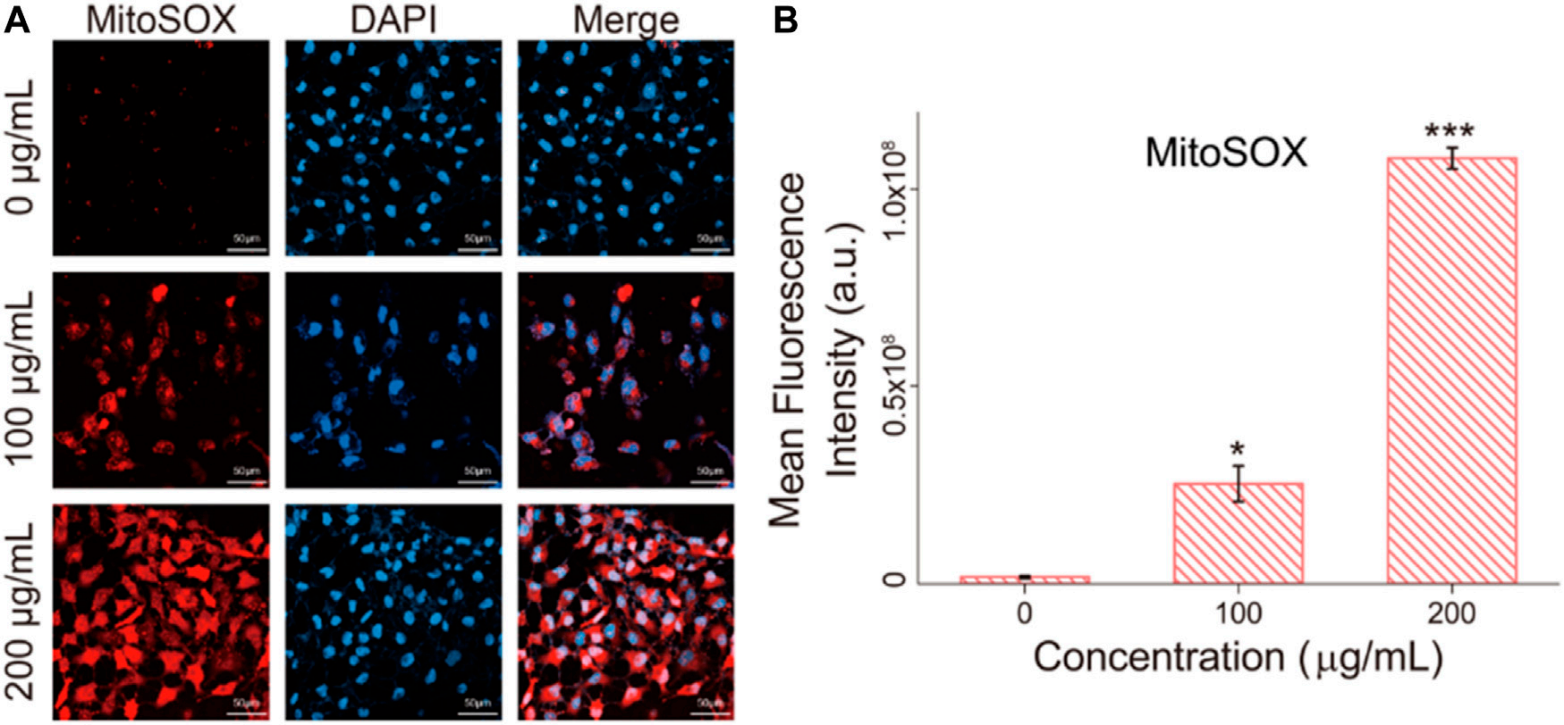
**(A)** mitochondrial superoxide levels in HBE cells were detected by Mitosox red after co-incubation with different concentration of Fe_3_O_4_/GO after 24 h (scale bar: 50 μm). **(B)** Mean fluorescence intensity of Mitosox red after co-incubation for 24 h.

Mitochondrial dysfunction was verified by Mitosox red, which is a mitochondrial superoxide indicator (Wang et al., 2013). Mitochondria are stimulated to produce mitochondrial superoxide, which leads to impaired mitochondrial function. As shown in Figure 7, after Fe_3_O_4_/GO exposure, red fluorescent was enhanced with concentration increased, which indicated mitochondrial generated superoxide. We can infer that ER-Ca^2+^ may induce mitochondrial dysfunction, after Fe_3_O_4_/GO exposure.

### Apoptosis of Fe_3_O_4_/GO Stimulation

**FIGURE 8:**
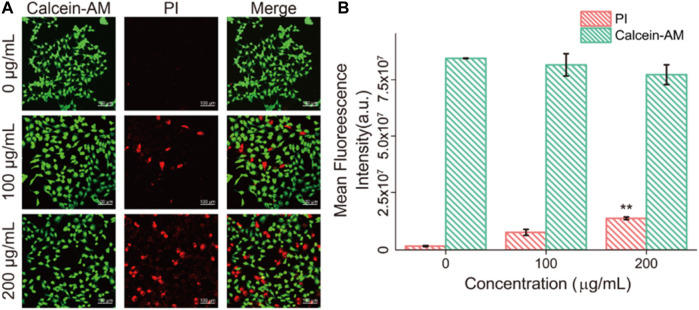
**(A)** HBE cells dead/live were detected by PI/Calcein-AM (red/green) after co-incubation with different concentration of Fe_3_O_4_/GO after 24 h (scale bar: 100 μm). **(B)** Mean fluorescence intensity of PI/Calcein-AM after co-incubation for 24 h.

The state of dead and alive cells was verified by calcein-AM/PI staining, where calcein-AM represented living cells (green fluorescent) and PI (red fluorescent) represented dead cells (Zheng et al., 2020). Figure 8 showed that after 24 h coincubation with Fe_3_O_4_/GO, part of the HBE cells died and the amount of dead cells response to increased Fe_3_O_4_/GO concentration.

**FIGURE 9:**
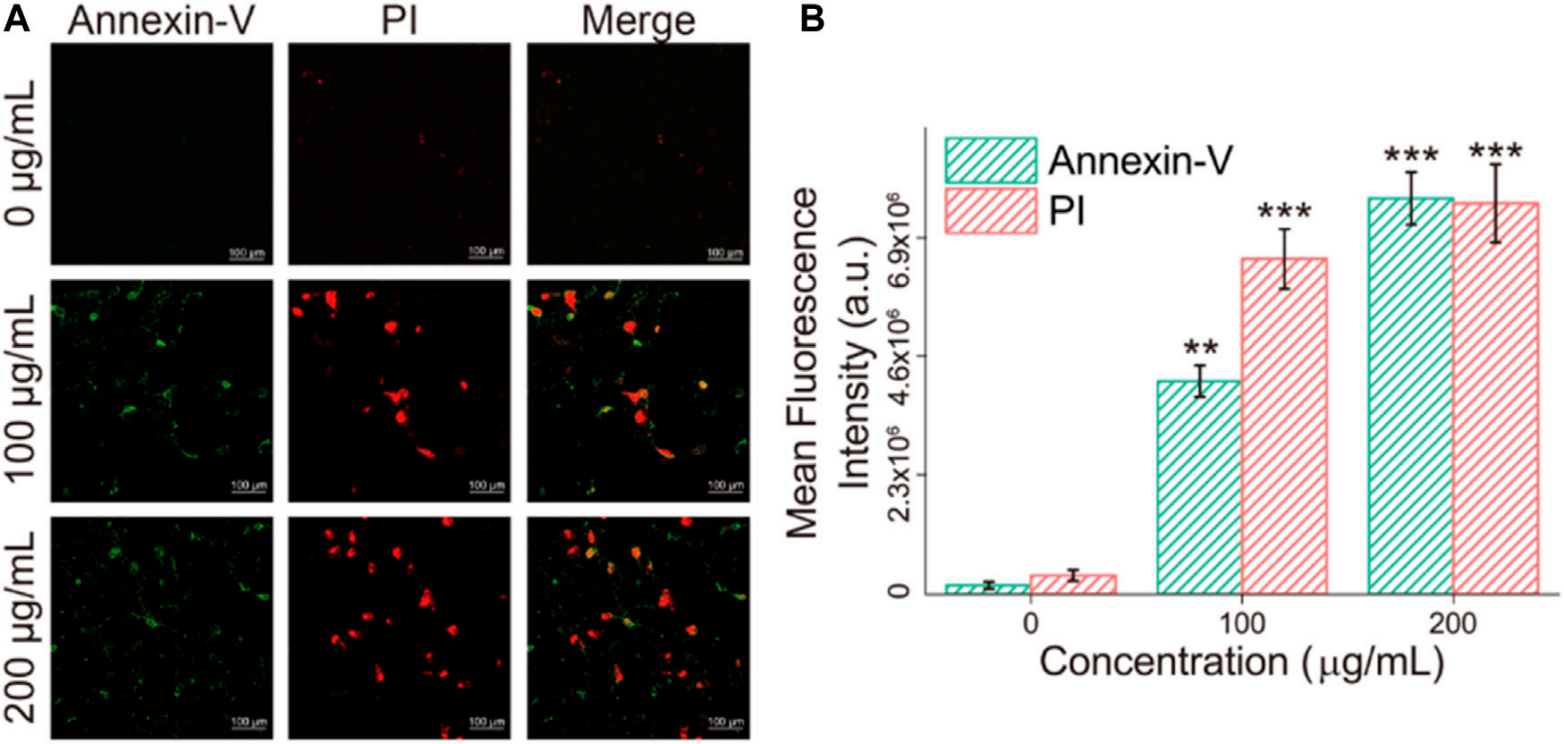
**(A)** HBE cells apoptosis were detected by Annexin-V/PI (green/red) after co-incubation with different concentration of Fe_3_O_4_/GO after 24 h (scale bar: 100 μm). **(B)** Mean fluorescence intensity of Annexin-V/PI (green/red) after co-incubation after 24 h.

Apoptosis detection was performed to verify the death mode of cells after incubation with Fe_3_O_4_/GO, which was characterized via Annexin-V/PI in Figure 9. After Fe_3_O_4_/GO exposure, fluorescence of Annexin-V and PI were obviously increased response to Fe_3_O_4_/GO nanoparticle concentration, which were concordant with the results of the cck-8 assay. It indicated high concentration Fe_3_O_4_/GO can lead to HBE cell apoptotic (Chen M. et al., 2021).

So far, we have confirmed that Fe_3_O_4_/GO can stimulate HBE cells to produce oxidative stress, calcium influx, and mitochondrial superoxide, ultimately leading to apoptosis, and these phenomena are concentration-dependent. In order to explore the specific pathway through which Fe_3_O_4_/GO stimulates HBE cell genesis, we characterized the expression levels of Caspase-9 and Caspase-3.

### Caspase-9/Caspase-3 Activated *via* Mitochondrial Damage

**FIGURE 10:**
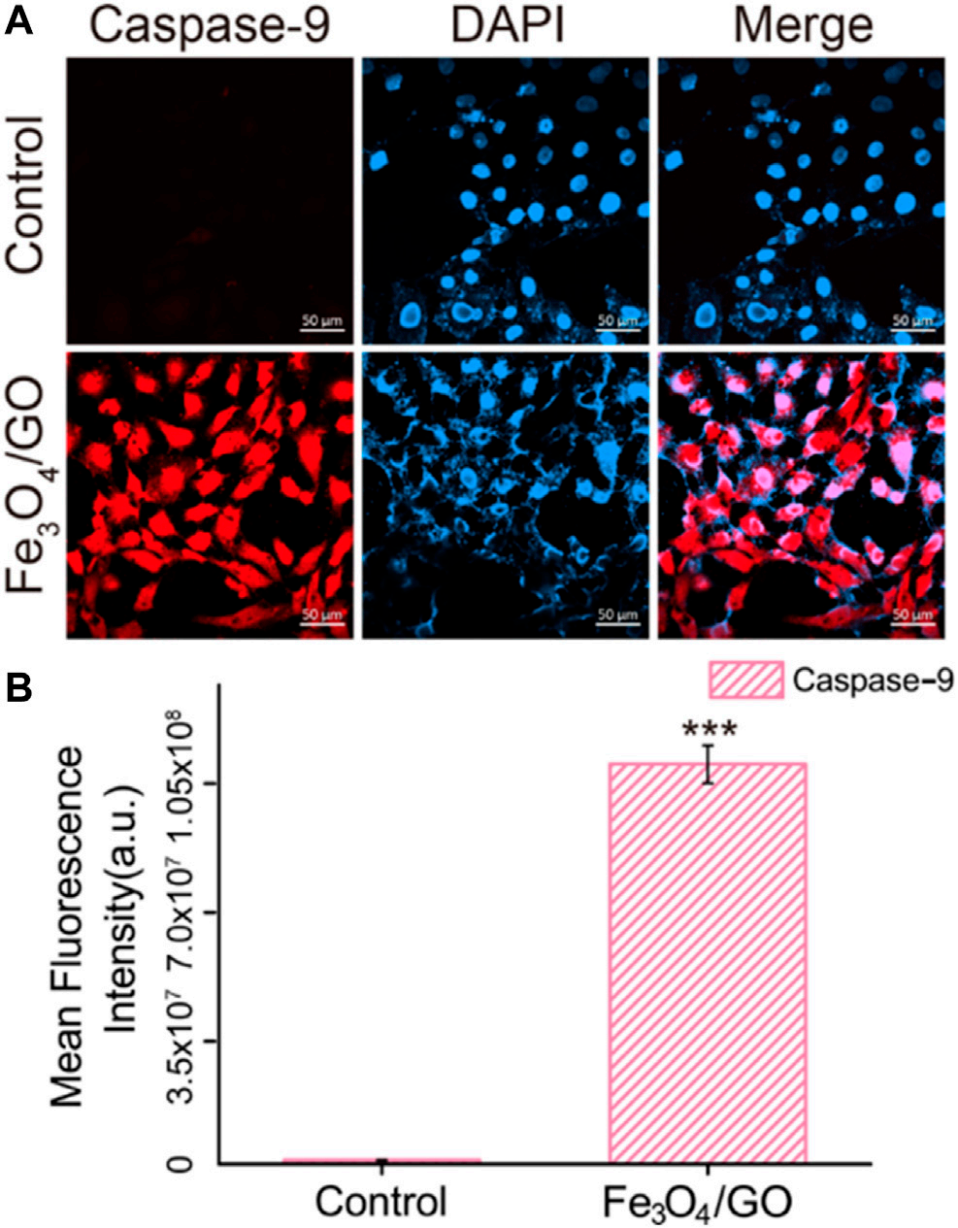
**(A)** Immunefluorescence of Caspase-9 after Fe_3_O_4_/GO exposure. (scale bar: 50 μm) **(B)** Mean fluorescence intensity of Caspase-9 after Fe_3_O_4_/GO nanoparticle exposure.

**FIGURE 11:**
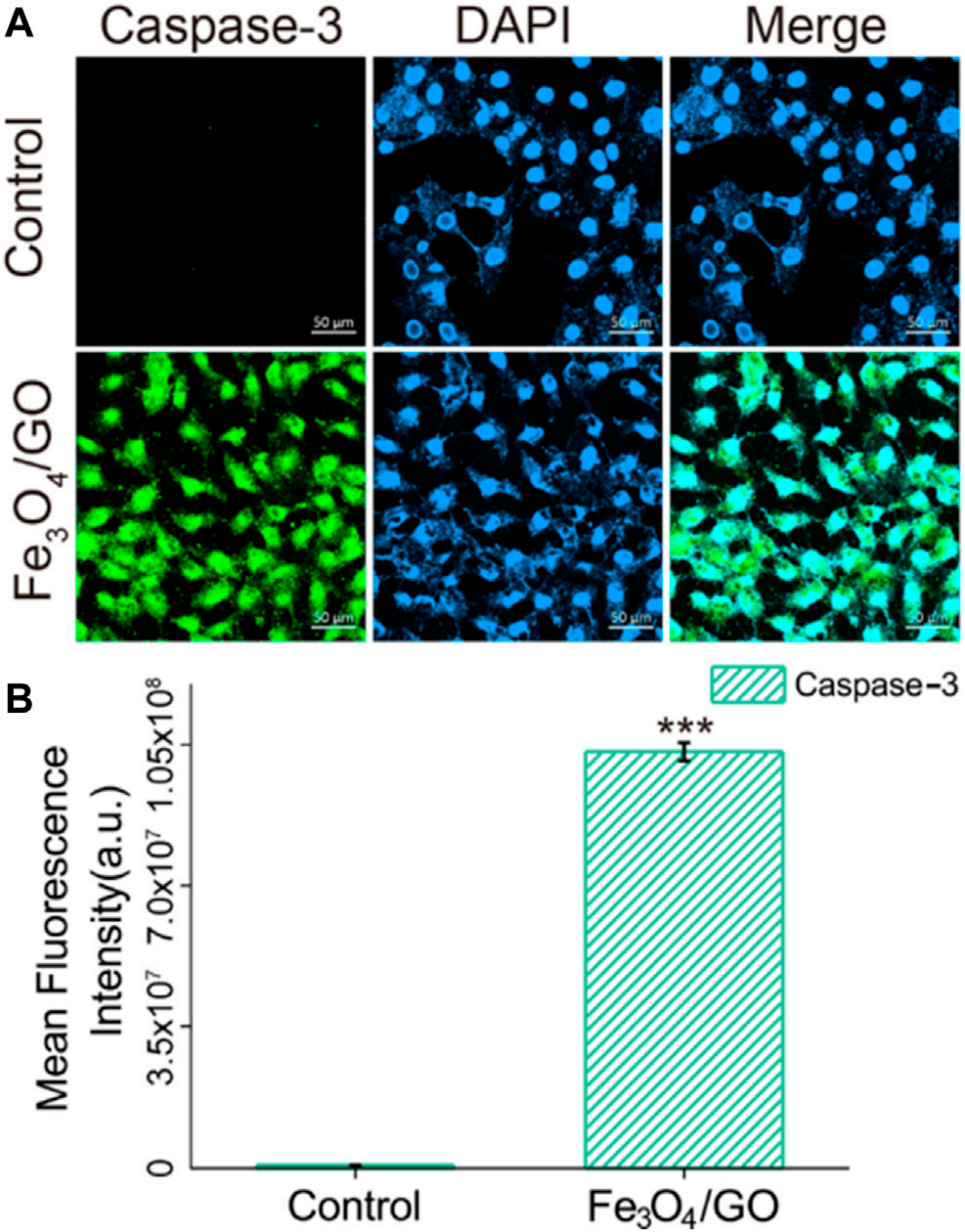
**(A)** Immunefluorescence of Caspase-3 after Fe_3_O_4_/GO exposure. (scale bar: 50 μm) **(B)** Mean fluorescence intensity of Caspase-3 after Fe_3_O_4_/GO exposure.

After coincubation with Fe_3_O_4_/GO, the fluorescence of Caspase-9 and Caspase-3 was significantly enhanced in Figures 10, 11, confirming that both Caspase-9 and Caspase-3 were activated. All these results indicated that Ca^2+^-ER stress led to mitochondrial dysfunction, which promoted the activation of Caspase-9 and activation of Caspase-3, resulted in cell apoptosis (Chen et al., 2005).

**FIGURE 12:**
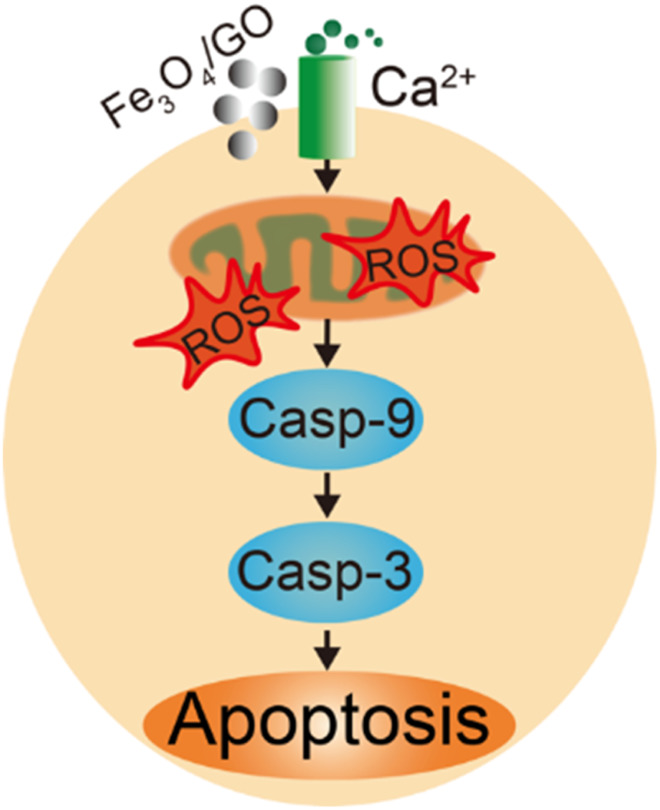
Scheme of HBE cells apoptosis pathway after Fe_3_O_4_/GO stimulation.

ROS are produced in cells via a variety of mechanisms. High intracellular ROS increased Ca^2+^ levels, which can trigger a series of mitochondrial related events, including endoplasmic reticulum stress, and mitochondrial dysfunction, then activated Caspase-9/Caspase-3 relate apoptosis, these proteins are important in apoptotic pathway (Figure 12) (Hailan et al., 2022). Our study demonstrated that after HBE cells co-incubation with Fe_3_O_4_/GO, ROS levels increased and Ca^2+^ levels enhanced, lead to mitochondrial dysfunction, and then, resulting in Caspase-9/Caspase-3 related apoptotic of HBE cells.

## Conclusion

In this study, the obtained results elaborated the cytotoxicity effects of Fe_3_O_4_/GO. Specifically, after exposure to high concentration of Fe_3_O_4_/GO nanomaterials, ROS levels and Ca^2+^ influx enhanced, and then, mitochondrial dysfunction, thereby leading to cell apoptosis *via* the Caspase-9/Caspase-3 pathway ultimately. The results also demonstrated that the cytotoxicity of Fe_3_O_4_/GO was in time-dependent and concentration-dependent manners. Therefore, it is still a challenging task in the future to transform Fe_3_O_4_/GO nanocomposites with cytotoxicity into biocompatible Fe_3_O_4_/GO nanocomposites.

## Data Availability

The original contributions presented in the study are included in the article/Supplementary Material, further inquiries can be directed to the corresponding authors.
