# Supplementary material for: Cytotoxicity Effect of Iron Oxide (Fe3O4)/Graphene Oxide (GO) Nanosheets in Cultured HBE Cells
Source: Front Chem. 2022 May 9;10:888033. doi: 10.3389/fchem.2022.888033 (PMC9124895; doi:10.3389/fchem.2022.888033)
Supplement: Supplementary file 1 [file DataSheet1.docx]

**SUPPLEMENTARY MATERIAL**

**Cytotoxicity Effect of Iron Oxide (Fe_3_O_4_)/ Graphene Oxide (GO) Nanosheets in Cultured HBE Cells**

Zhang Yule^1, #^, Zhang Yatian^2, #^, Yang Zhijin^1^, Fan Yan^1^, Chen Mengya^1^, Zhao Mantong^4^, Dai Bo^1^, Zheng Lulu^1, *^, Zhang Dawei^1,3*^

^1^ Engineering Research Center of Optical Instrument and System, the Ministry of Education, Shanghai Key Laboratory of Modern Optical System, University of Shanghai for Science and Technology, Shanghai 200093, P.R. China；

^2^ Medical College Jining Medical University, Jining Shandong 272067, P.R. China ；

^3^ Shanghai Institute of Intelligent Science and Technology, Tongji University, Shanghai 200092, P.R. China;

^4^ Department of Physics and Electronic Engineering, Heze University, Heze 274015, P.R. China.

^#^ These authors are contributed equally to this work.

^*^ Correspondence: [llzheng@usst.edu.cn](mailto:llzheng@usst.edu.cn), dwzhang@usst.edu.cn


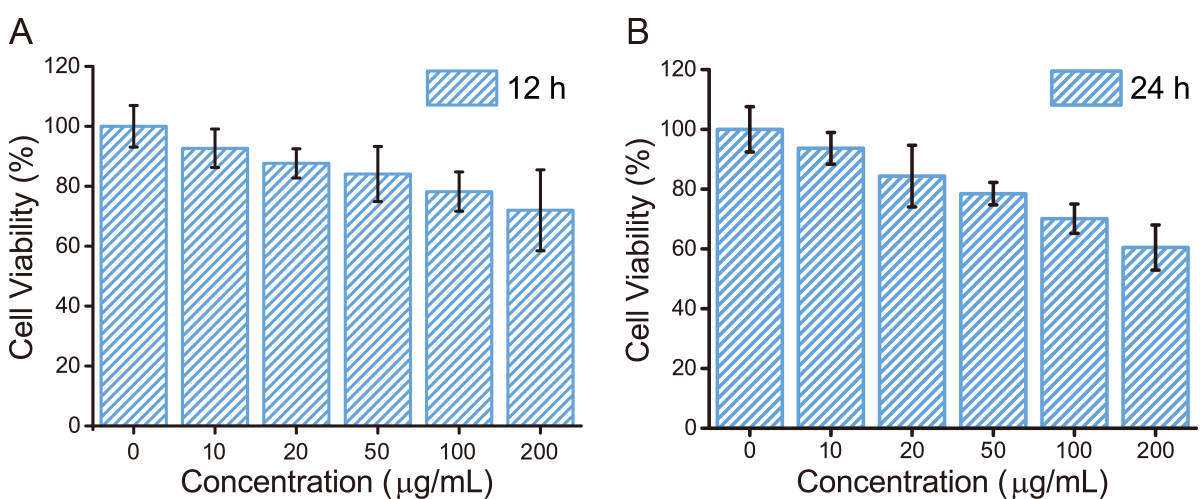


Figure S1. A-B) Cell viability of BEAS-2B cells after co-incubated with different concentration of Fe_3_O_4_/GO after 12 and 24 h, respectively.
